# Renal abnormalities in rheumatoid arthritis: an insight on IgA nephropathy

**DOI:** 10.1093/rap/rkab109

**Published:** 2021-12-27

**Authors:** Ana R Prata, Helena Assunção, Gisela Eugénio, Vítor Sousa, Cátia Duarte

**Affiliations:** 1 Rheumatology Unit, Hospitais da Universidade de Coimbra—Centro Hospitalar e Universitário de Coimbra, Coimbra; 2 Rheumatology Unit, Centro Hospitalar do Baixo Vouga, Aveiro; 3 Pathological Anatomy Unit, Hospitais da Universidade de Coimbra—Centro Hospitalar e Universitário de Coimbra; 4 Institute of Pathological Anatomy; 5 Coimbra Institute for Clinical and Biomedical Research (iCBR), Faculty of Medicine, University of Coimbra, Coimbra, Portugal

Key message

- IgA nephropathy is an uncommon differential diagnosis of proteinuria in RA.

Dear Editor, Renal abnormalities are relatively common in RA [1] and require careful clinical and laboratory evaluation and consideration of a broad spectrum of differential diagnoses. Histopathological lesions in RA kidney biopsies include several glomerular and tubulointerstitial pathological entities, which are most frequently attributed to the use of nephrotoxic drugs, such as NSAIDs and some conventional synthetic DMARDs [2]. Nevertheless, the diagnosis of renal amyloidosis and other probably disease-related nephropathies has also been reported in RA patients [3].

We report the case of a previously healthy 51-year-old male who presented with a 12-month history of symmetrical polyarthritis. Laboratory findings revealed extremely high titres of RF [2190 IU/mL (normal <20 IU/mL)], ACPA [> 600 IU/mL (normal <7 IU/mL)] and elevated CRP [2.22 mg/dL; (normal < 0.50 mg/dL)] and ESR [72 mm/h; (normal <20 mm/h)]. Plain radiographs revealed erosions in several MCP joints. The patient was diagnosed with RA and started on oral MTX 15 mg/week, oral prednisolone 7.5 mg/day, and naproxen 500 mg, as needed, for pain relief. After 3 months, as high active disease persisted (DAS-28–ESR 5.18), the dose of MTX was increased to 20 mg/week, and LEF 15 mg/day was added. At the same time, persistent proteinuria in a spot urine sample was identified and confirmed in 24 h urine sample analysis, reaching 2481.8 mg/24 h. Urinary sediment examination revealed haematuria (five red blood cells per high power field). The estimated glomerular filtration rate (EGFR) was within the normal range [modification of diet in renal disease study (MDRD) 101 mL/min/1.73 m^2^], and no hypertension or peripheral oedema was identified upon examination. Treatment with naproxen, MTX and LEF was stopped, and the patient was referred for kidney biopsy, which revealed an IgA nephropathy (Fig. 1). Rituximab was initiated for controlling RA disease activity, along with supportive renal treatment with perindopril 10 mg/day. Renal function remained preserved (EGFR 89.1 mL/min/1.73 m^2^), proteinuria decreased (547 mg/g in a spot urine sample), and no microscopic haematuria was detected at the last follow-up.

**Fig. 1:**
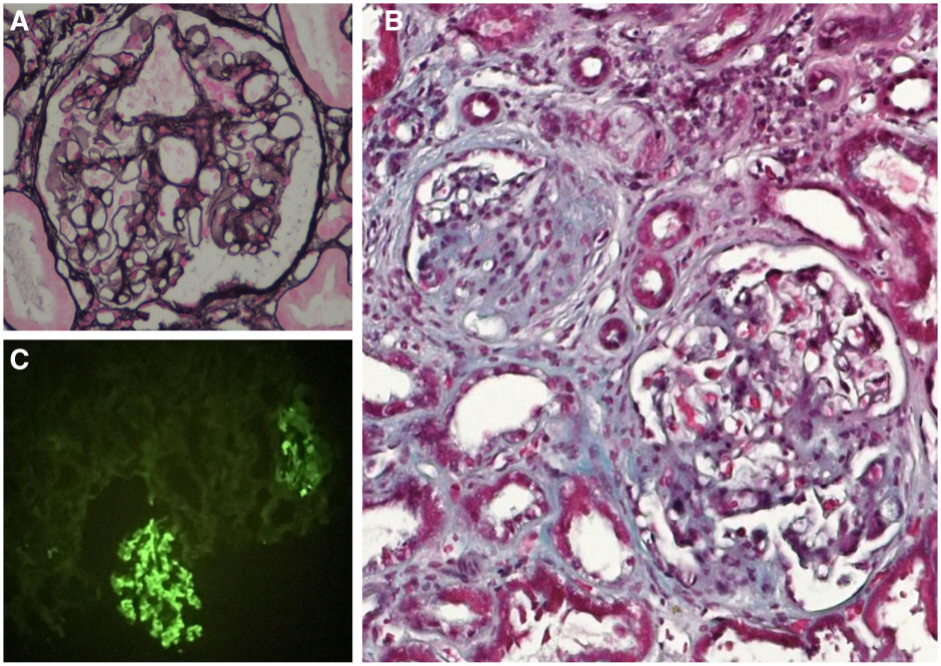
Light microscopy and immunofluorescence of the kidney biopsy specimen (**A**) Glomerulus with thin capillary walls, no double contour or spikes. Marinozzi stain, ×400. (**B**) One glomerulus (right part of the image) with areas of mesangial expansion and hypercellularity; a second glomerulus with a fibrocellular crescent is seen (left part of the image). Tubular atrophy and interstitial fibrosis are also observed. Masson’s Trichrome stain, ×200. (**C**) Immunofluorescence revealing mesangial deposits of IgA 2+. IgA, ×200.

IgA nephropathy is an infrequent renal complication in RA [4] that has been seldom documented in cohort studies [2, 5]. The pathophysiology of RA-associated IgA nephropathy remains poorly understood, although it is thought to be related to abnormalities in the glycosylation pattern of RF, leading to the deposition of immune complexes with galactose-deficient IgA1 on the glomeruli, resulting in renal damage [6, 7]. There are no clinical or histological features that can accurately distinguish primary IgA nephropathy from its secondary forms, usually related to systemic autoimmune diseases, such as RA; thereby, a presumptive diagnosis of secondary IgA nephropathy is typically made in the simultaneous presence of the two conditions. The treatment of primary IgA nephropathy is mainly supportive, whereas treatment in RA-associated IgA nephropathy should be based on controlling the activity of the underlying disease [7]. The prognosis of IgA nephropathy is highly variable but mostly linked to a high risk of renal failure [8].

This case highlights the need for continued monitoring of renal disease in RA patients through routine urinary analysis and clinical follow-up. Special attention must be given to patients with high disease activity under conventional synthetic DMARD and NSAID treatment, considering the possible nephrotoxicity of these therapies and the unusual but potentially severe RA renal complications, such as IgA nephropathy.

*Funding*: No specific funding was received from any bodies in the public, commercial or not-for-profit sectors to carry out the work described in this article.

*Disclosure statement:* The authors have declared no conflicts of interest.

## Data availability statement

The data underlying this article are available in the article.
